# Longitudinal Changes in Ganglion Cell Complex Thickness Before and After Intravitreal Bevacizumab Injections in Diabetic Macular Edema

**DOI:** 10.3390/diagnostics16182892

**Published:** 2026-09-08

**Authors:** Hye Won Jun, Daniel Duck-Jin Hwang

**Affiliations:** 1 Department of Ophthalmology, Hangil Eye Hospital, Incheon 21388, Republic of Korea; 2 Department of Ophthalmology, Catholic Kwandong University College of Medicine, Incheon 22711, Republic of Korea

**Keywords:** diabetic macular edema, ganglion cell complex, optical coherence tomography, retinal imaging

## Abstract

**Background/Objectives**: Diabetic macular edema (DME) is a major cause of vision impairment in patients with diabetes mellitus, and intravitreal anti-vascular endothelial growth factor (anti-VEGF) therapy is widely used for its management. However, the longitudinal effects of anti-VEGF treatment on the ganglion cell complex (GCC) remain unclear. This study evaluated changes in GCC thickness in eyes with DME following intravitreal bevacizumab injections over a one-year period. **Methods**: In this retrospective observational study, 55 eyes from 55 patients with DME who received intravitreal bevacizumab injections were analyzed. Central foveal thickness (CFT), total macular volume (TMV), and retinal layer thicknesses including the retinal nerve fiber layer (RNFL), ganglion cell layer (GCL), inner plexiform layer (IPL), and GCC were measured using spectral-domain optical coherence tomography and compared with those of fellow eyes without DME. **Results**: At baseline, eyes with DME showed significantly greater CFT, TMV, RNFL, IPL, and GCC thickness compared with fellow eyes. After one year of treatment, CFT and TMV decreased significantly, and significant reductions were also observed in RNFL, GCL, IPL, and GCC thickness. At the one-year follow-up, GCC and inner retinal layer thicknesses were no longer significantly different between DME and fellow eyes. Multiple regression analysis showed that final visual acuity was associated with baseline visual acuity and CFT at one year, but not with GCC thickness. **Conclusions**: These findings suggest that the reduction in GCC thickness after bevacizumab treatment may reflect anatomical normalization following edema resolution rather than true ganglion cell loss. Further studies with larger cohorts and longer follow-up are required to clarify the long-term effects of anti-VEGF therapy on retinal neurostructure.

## 1. Introduction

Diabetic retinopathy (DR) is a significant microvascular complication of diabetes mellitus, affecting both type 1 and type 2 diabetes. Diabetic macular edema (DME) is the leading cause of vision impairment and legal blindness among individuals with diabetes [1,2,3]. However, the precise pathophysiology underlying the development of DME is not yet entirely understood. It is hypothesized that disruption of the blood–retinal barrier, resulting in fluid accumulation within the macular layers, plays a central role in the onset of DME [4]. Among several factors involved in the pathogenesis of DME, vascular endothelial growth factor (VEGF) is a key molecular mediator, contributing to increased vascular permeability and inflammation [5,6]. Consequently, intravitreal anti-VEGF therapy has become one of the most widely accepted and effective treatments for DME [7,8,9,10,11].

Beyond vascular abnormalities, increasing evidence suggests that diabetic retinal neurodegeneration is an important component of diabetic retinal disease and may occur even before clinically detectable microvascular changes [12]. This neurodegenerative process predominantly affects the inner retinal layers, including the RNFL, GCL, and IPL [13]. Structural alterations of these inner retinal layers have been reported to be more pronounced in eyes with DME than in diabetic eyes without macular edema [14]. However, because retinal edema itself influences OCT-derived measurements of the inner retinal layers, it remains unclear whether longitudinal changes in GCC thickness after anti-VEGF treatment reflect progressive retinal neurodegeneration or anatomical normalization associated with edema resolution. Addressing this question is clinically important because accurate interpretation of GCC thickness may improve the assessment of retinal neuronal damage and disease progression in patients with DME.

Taken together, these observations highlight the need for longitudinal evaluation of GCC thickness in eyes with DME receiving anti-VEGF therapy. Such evaluation may help distinguish edema-related anatomical normalization from true retinal neurodegeneration. Therefore, the purpose of this study was to investigate longitudinal changes in GCC thickness over one year following intravitreal bevacizumab treatment.

## 2. Materials and Methods

The Institutional Review Board of Hangil Eye Hospital approved this retrospective study (IRB No. 25009). All study procedures complied with the principles of the Declaration of Helsinki. Written informed consent was waived owing to the retrospective nature of the study.

### 2.1. Patients

This retrospective observational case series included consecutive patients treated at Hangil Eye Hospital between February 2015 and December 2021. A total of 55 eyes from 55 patients with DME who received intravitreal bevacizumab injections were enrolled. Eligible participants were required to be older than 20 years and to have diabetic retinopathy with OCT-confirmed DME. DME was defined as a central foveal thickness (CFT) exceeding 320 μm on optical coherence tomography (OCT).

Eyes with retinal diseases other than diabetic retinopathy, including retinal vein occlusion, epiretinal membrane, vitreomacular traction, and age-related macular degeneration, were excluded. Patients with glaucoma, uveitis, or other vitreoretinal disorders were also excluded. Additional exclusion criteria included previous focal or grid laser photocoagulation, panretinal photocoagulation, intravitreal treatment for DME within the previous six months, ocular trauma, and any history of ocular surgery other than cataract extraction.

The date of the first intravitreal bevacizumab injection was designated as the baseline visit. Thereafter, retreatment was performed using a pro re nata (PRN) regimen according to the treating physician’s clinical judgment. Clinical records and spectral-domain OCT (SD-OCT) images obtained at baseline, each injection visit, and 12 months after the initial injection were reviewed. The control group consisted of the non-DME fellow eyes of the enrolled patients, which met the same inclusion criteria but showed no signs of DME during the 1-year follow-up period. Fellow eyes were selected as controls to minimize interindividual variability and potential confounding by systemic factors, as both eyes were exposed to the same systemic diabetes mellitus. The severity of diabetic retinopathy was comparable between the study and control eyes. However, these fellow eyes were not considered normal controls, as diabetes-related neurodegenerative changes may occur bilaterally even in the absence of DME.

### 2.2. Optical Coherence Tomography Scanning Protocols

Spectral-domain optical coherence tomography (SD-OCT) imaging was performed using the Spectralis OCT system (Heidelberg Engineering, Heidelberg, Germany). Each examination included a macular volume scan composed of 25 horizontal raster scans centered on the fovea, covering a 6 × 6 mm retinal area. These scans were used to obtain retinal thickness measurements and volumetric data. All OCT examinations were performed using the same device and standardized acquisition protocol throughout the study period, thereby minimizing inter-scan variability and avoiding inter-device variability.

Central foveal thickness (CFT) was defined as the mean retinal thickness within the central 1 mm subfield of the Early Treatment Diabetic Retinopathy Study (ETDRS) map. Total macular volume (TMV) was automatically generated by Heidelberg Eye Explorer software (version 1.10.2.0; Heidelberg Engineering GmbH, Heidelberg, Germany) for the entire 6 mm ETDRS circle.

Automated segmentation of retinal layers was performed using the same software. The ganglion cell complex (GCC) was defined as the combination of the retinal nerve fiber layer (RNFL), ganglion cell layer (GCL), and inner plexiform layer (IPL). Each raster scan was individually reviewed, and any segmentation errors identified in the RNFL, GCL, or IPL boundaries were manually corrected before quantitative analysis. Thus, GCC quantification was based on the established segmentation algorithm implemented in the Heidelberg Eye Explorer software rather than on a novel segmentation methodology.

To avoid potential bias, RNFL, GCL, and IPL thicknesses were not evaluated within the central 1 mm ETDRS subfield. Instead, average layer thicknesses were derived from the corresponding retinal volumes measured within the entire 6 mm ETDRS circle using the following equation:

Average thickness (μm) = [Macular volume of 6 mm circle (mm^3^)/(9π)] × 1000

Thickness of layers were measured before and after the initial bevacizumab injection, and the values were compared with time progression in the DME group.

### 2.3. Intravitreal Injection Methods

Before the injection, topical anesthesia was induced with 0.5% proparacaine. The ocular surface was prepared with 5% povidone–iodine, after which sterile draping and lid speculum placement were performed. Under sterile conditions, 1.25 mg/0.05 mL of bevacizumab was injected through the pars plana at the baseline visit and at subsequent retreatment visits. Upon completion of the injection, the entry site was gently compressed with a sterile cotton applicator. Topical antibiotic eye drops were prescribed for three days, with instructions to instill them four times daily.

### 2.4. Statistical Analysis

Statistical analysis was performed using the IBM SPSS Statistics program, with statistical significance defined as *p* < 0.05. The normality of paired differences was assessed using the Shapiro–Wilk test and Q–Q plots. Paired *t*-tests were used when the normality assumption was satisfied, whereas the Wilcoxon signed-rank test was used for paired comparisons that did not meet the normality assumption.

For longitudinal analyses, repeated-measures analysis of variance (ANOVA) was performed to evaluate changes in retinal layer thicknesses over time. The distribution of retinal layer thickness measurements at each follow-up time point was assessed using the Shapiro–Wilk test and Q–Q plots. Because the assumption of sphericity was violated, Greenhouse–Geisser correction was applied. Bonferroni-adjusted pairwise comparisons were performed between baseline and each follow-up visit.

To identify factors associated with final visual acuity (VA), a multiple linear regression analysis was performed. The model included baseline VA, age, sex, CFT, and RNFL, GCL, and IPL thicknesses measured at baseline and at the 12-month follow-up as independent variables. The assumptions of multiple linear regression were assessed using residual diagnostic plots and the Shapiro–Wilk test. No substantial violations of normality, linearity, or homoscedasticity were observed.

## 3. Results

### 3.1. Baseline Characteristics

A total of 55 eyes from 55 patients with DME were included in this study. The mean age was 53.47 ± 9.78 years, and 63.6% of patients were male. The mean duration of diabetes mellitus was 11.40 ± 7.51 years, with a mean HbA1c level of 8.34 ± 1.80%. Most eyes were phakic (76.4%), and the mean spherical equivalent was −0.60 ± 2.00 diopters. The mean baseline best-corrected visual acuity (BCVA) was 0.32 ± 0.20 logMAR (Table 1). The patients received a mean of 3.6 ± 2.0 (range: 1–8) intravitreal bevacizumab injections.

### 3.2. Baseline Comparison Between DME Eyes and Fellow Eyes

At baseline, DME eyes demonstrated significantly greater central foveal thickness (CFT) and total macular volume (TMV) compared with non-DME fellow eyes (both *p* < 0.001). The mean GCC thickness was significantly higher in DME eyes than in fellow eyes (127.97 ± 19.41 μm vs. 109.13 ± 15.04 μm, *p* = 0.027). Similarly, RNFL thickness and IPL thickness were significantly increased in DME eyes (*p* = 0.005 and *p* = 0.042, respectively). In contrast, GCL thickness did not differ significantly between the two groups at baseline (*p* = 0.628) (Table 2).

### 3.3. Longitudinal Changes over 12 Months

BCVA significantly improved from 0.32 ± 0.20 logMAR at baseline to 0.24 ± 0.22 logMAR at 12 months (*p* = 0.006), whereas BCVA in fellow eyes remained unchanged from baseline to 12 months (0.09 ± 0.10 logMAR at both time points, *p* = 1.000). At 12 months, BCVA did not differ significantly between DME and fellow eyes (*p* = 0.073) (Table 3). After one year of intravitreal bevacizumab treatment, DME eyes showed significant reductions in CFT and TMV compared with baseline (both *p* < 0.001). GCC thickness also significantly decreased from 127.97 ± 19.41 μm at baseline to 117.09 ± 20.88 μm at 12 months (*p* < 0.001). Significant thinning was observed in RNFL, GCL, and IPL thicknesses over the same period based on paired comparisons between baseline and 12 months (*p* < 0.001, *p* < 0.001, and *p* = 0.006, respectively).

**Figure 1:**
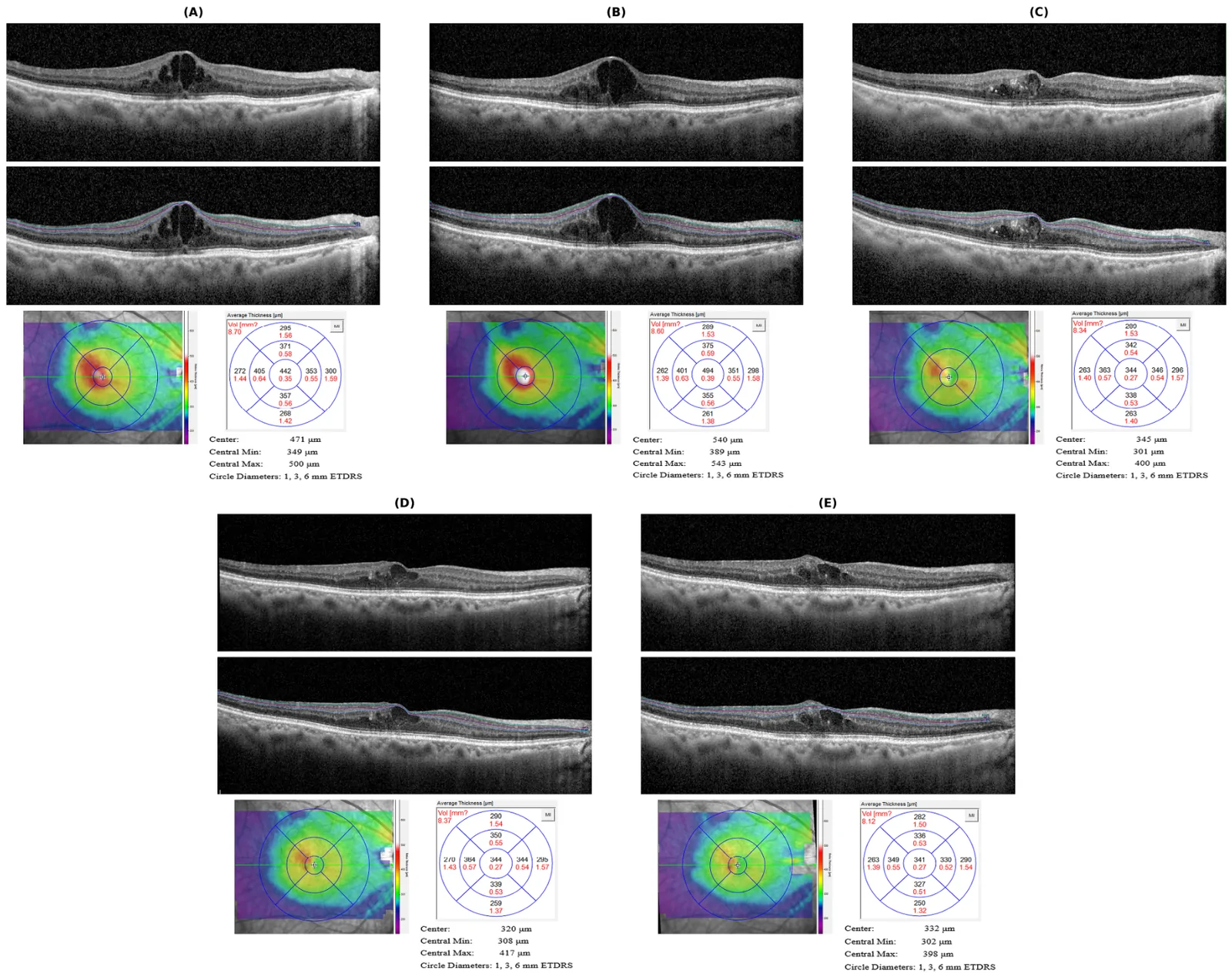

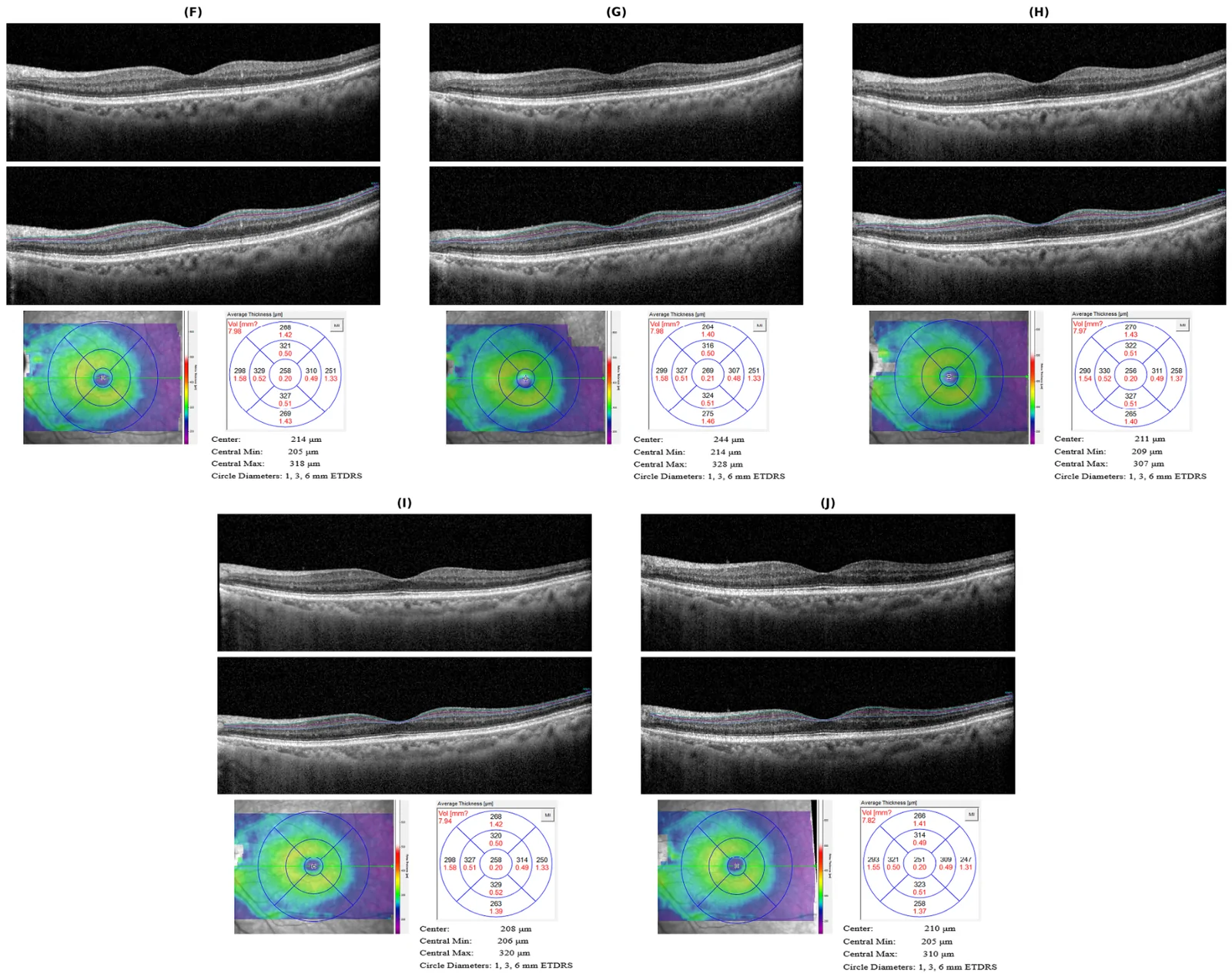
Representative optical coherence tomography (OCT) images with diabetic macular edema (DME) and the non-DME fellow eye. OCT images of the DME eye were obtained at baseline (**A**), 1 month (**B**), 3 months (**C**), 6 months (**D**), and 12 months (**E**) after the first intravitreal bevacizumab injection. Corresponding OCT images of the non-DME fellow eye from the same patient were obtained at baseline (**F**), 1 month (**G**), 3 months (**H**), 6 months (**I**), and 12 months (**J**). Each panel includes the retinal thickness map, horizontal B-scan OCT image, and the corresponding retinal layer segmentation image used for ganglion cell complex (GCC) measurements. The segmentation images from top to bottom represent the retinal nerve fiber layer (RNFL), ganglion cell layer (GCL), and inner plexiform layer (IPL), respectively, with the colored lines indicating the boundaries of each segmented retinal layer. Progressive resolution of macular edema in the treated DME eye was accompanied by restoration of retinal architecture and anatomical normalization of the inner retinal layers throughout the follow-up period.

**Figure 2:**
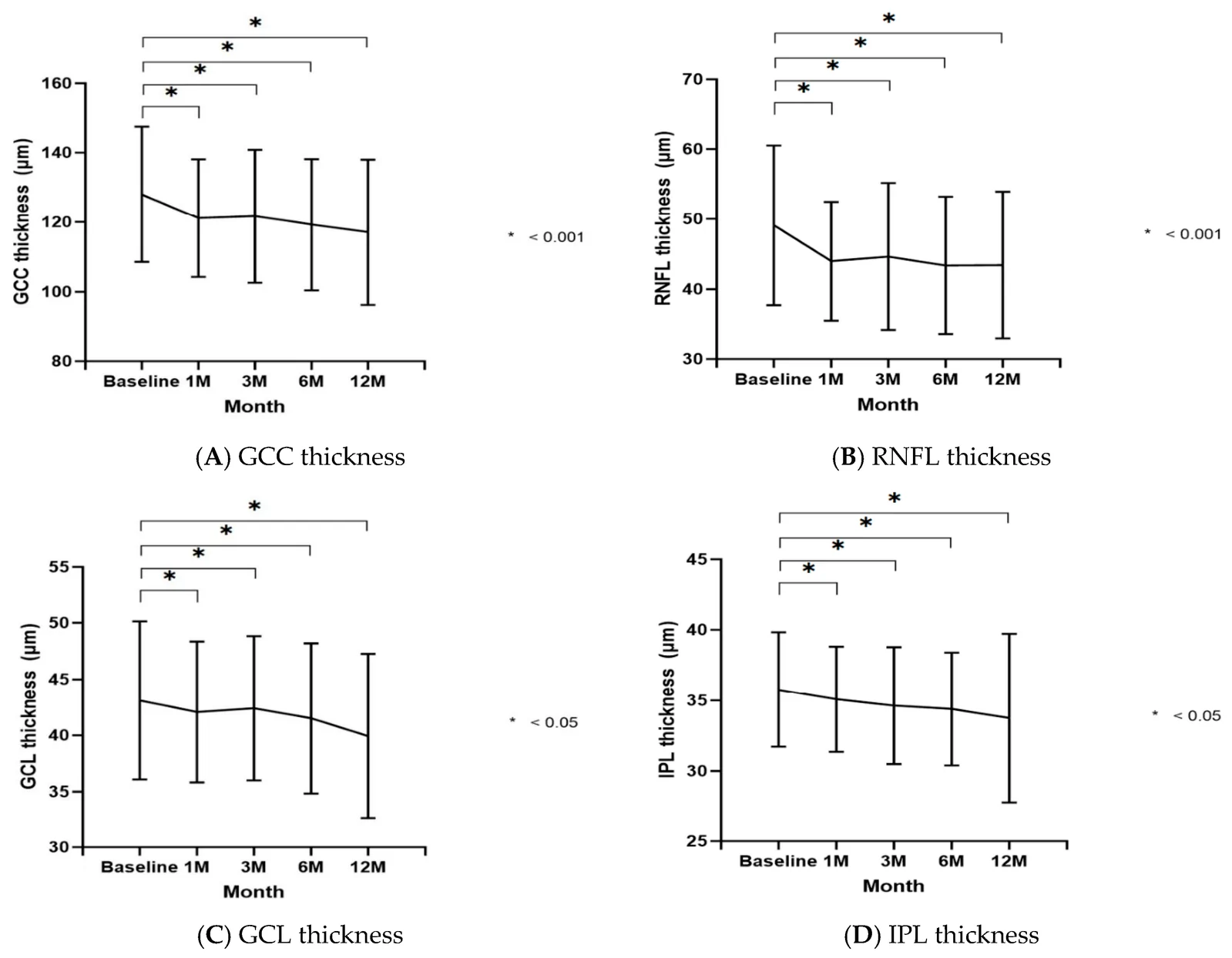
Longitudinal changes in (**A**) GCC, (**B**) RNFL, (**C**) GCL, and (**D**) IPL thickness following intravitreal bevacizumab treatment. Retinal layer thicknesses were measured at baseline and at 1, 3, 6, and 12 months after the first injection. The number of eyes included at baseline and at 1, 3, 6, and 12 months was 55, 40, 44, 44, and 55, respectively. Repeated-measures ANOVA with Greenhouse-Geisser correction was used to assess longitudinal changes. Pairwise comparisons between baseline and each follow-up visit were conducted using Bonferroni adjustment. Bonferroni-adjusted *p* < 0.05 compared to baseline is indicated by asterisks. Standard deviations are shown by error bars.

Repeated-measures ANOVA with Greenhouse–Geisser correction demonstrated significant longitudinal changes over time in GCC (F = 23.929, *p* < 0.001), RNFL (F = 17.847, *p* < 0.001), GCL (F = 15.872, *p* < 0.001), and IPL (F = 14.305, *p* < 0.001) thicknesses. Representative serial OCT images demonstrate progressive resolution of macular edema, restoration of retinal architecture, and corresponding changes in GCC segmentation throughout the follow-up period (Figure 1). Bonferroni-adjusted pairwise comparisons demonstrated that GCC, RNFL, GCL, and IPL thicknesses were significantly reduced at all follow-up visits compared with baseline (Figure 2).

When compared with fellow eyes at 12 months, DME eyes no longer showed significant differences in GCC, RNFL, GCL, or IPL thickness (all *p* > 0.05). However, CFT and TMV remained significantly greater in DME eyes than in fellow eyes (*p* <0.001 and *p* = 0.013, respectively) (Table 3). Although CFT, TMV, and GCC thickness showed slight numerical decreases in fellow eyes from baseline to 12 months, these changes were not statistically significant (all *p* > 0.05).

### 3.4. Correlation Analysis of Factors Affecting Final BCVA

Final visual acuity was substantially correlated with baseline BCVA (β = 0.528, *p* < 0.001) and CFT at 12 months (β = 0.326, *p* = 0.025), according to multiple regression analysis. Final visual acuity outcomes were not significantly correlated with age, sex, baseline CFT, or baseline or follow-up assessments of GCC sublayers (RNFL, GCL, and IPL) (Table 4).

## 4. Discussion

The principal finding of our study is that the reduction in GCC thickness following anti-VEGF treatment appears to reflect anatomical normalization associated with edema resolution rather than progressive ganglion cell loss. Although GCC thickness significantly decreased during follow-up, the absence of a significant difference between DME and fellow eyes at 1 year suggests that the initially increased GCC thickness was largely attributable to retinal edema rather than irreversible neurodegenerative changes.

Previous studies have shown conflicting results regarding peripapillary RNFL (pRNFL) thickness in DME. One study demonstrated thicker pRNFL measurements in DME eyes compared with non-DME eyes [15], whereas another found no significant difference between DME eyes and normal controls [16]. Notably, the latter study included eyes that had undergone panretinal photocoagulation (PRP), focal laser, or intravitreal injections, all of which may have contributed to reduced pRNFL thickness. To avoid such confounding factors, we excluded patients with a history of laser treatment or intravitreal injections. While prior studies have mainly focused on pRNFL thickness, our study specifically examined macular RNFL (mRNFL) thickness in DME eyes. We found that macular retinal nerve fiber layer (mRNFL) thickness was significantly greater in eyes with diabetic macular edema (DME) than in fellow eyes at baseline, consistent with findings from a previous study [17], although that study primarily compared RNFL thickness between DME eyes and healthy control eyes. In our study, mRNFL thickness significantly decreased at 1 year after bevacizumab treatment, a finding consistent with a previous study that reported a significant reduction in mRNFL thickness 1 year after aflibercept injections [18]. In our study, no significant difference in mRNFL thickness was observed between DME eyes and fellow eyes at the 1-year follow-up. These findings suggest that increased mRNFL thickness in DME likely reflects edema-related retinal swelling, which subsequently resolves following treatment.

Several studies have demonstrated controversial results in inner retinal layer thickness changes after intravitreal injections in DME eyes. In particular, GC–IPL thickness was shown to significantly decrease 12 months after aflibercept injection [18]. Another study reported an increase in GCC thickness one month after intravitreal bevacizumab injections [19]. Another study evaluating GCC thickness after three monthly ranibizumab injections found a significant reduction at 3 months, but no significant change at 6 months [20]. In contrast, our study demonstrated a sustained reduction in GCC thickness at 1 month, 3 months, 6 months, and 1 year after the first injection. This discrepancy may be explained by differences in treatment regimens, as eyes in the previous study did not receive additional injections after the initial three doses, whereas our study employed a pro re nata (PRN) regimen, potentially resulting in more sustained anatomical changes. Importantly, the absence of a significant difference in GCC thickness between DME and fellow eyes at 1 year suggests that the initially increased GCC thickness in DME eyes decreased to a level comparable to that of non-DME eyes following treatment. This pattern suggests GCC thickness decrease after bevacizumab treatment may represent anatomical normalization following edema resolution, rather than clinically meaningful ganglion cell loss.

Although diabetic retinal neurodegeneration is now recognized as an important component of diabetic retinal disease, interpretation of GCC thickness in eyes with DME requires particular caution. Previous studies have consistently demonstrated progressive thinning of the RNFL, GCL, and IPL in diabetic patients, suggesting that neurodegeneration begins before clinically apparent microvascular abnormalities and progresses throughout the course of diabetic retinopathy [12,21]. However, eyes with DME represent a distinct clinical condition because intraretinal fluid accumulation alters retinal architecture and substantially influences OCT-derived measurements of the inner retinal layers. Ahmad et al. demonstrated that total retinal thickness was the strongest determinant of macular GC-IPL thickness in diabetic eyes, explaining more than half of its variability, highlighting the significant influence of retinal edema on inner retinal thickness measurements [22]. Likewise, Hwang et al. reported that DME significantly increased peripapillary RNFL thickness, potentially confounding the structural evaluation of glaucoma [15]. Furthermore, Yang et al. demonstrated that the apparent reduction in RNFL thickness following anti-VEGF therapy was markedly attenuated after adjustment for retinal thickness, suggesting that edema resolution rather than true axonal loss accounted for much of the observed thinning [23]. Collectively, these studies suggest that reductions in inner retinal layer thickness after anti-VEGF therapy should be interpreted in the context of retinal edema rather than being regarded as direct evidence of progressive retinal neurodegeneration. It should be acknowledged that retinal edema is not the sole determinant of GCC thickness. Inner retinal layer measurements may also be influenced by diabetic neurodegeneration, retinal ischemia, aging, intraocular pressure, and other ocular or systemic factors. Therefore, although retinal edema substantially affects OCT-derived GCC measurements, longitudinal changes in GCC thickness are likely multifactorial and should be interpreted within the broader clinical context rather than being attributed to a single mechanism.

Our findings are consistent with this concept. At baseline, GCC thickness was significantly greater in DME eyes than in fellow eyes, whereas no significant difference remained after one year of anti-VEGF treatment despite a significant reduction in GCC thickness. If progressive ganglion cell loss had been the predominant mechanism underlying the observed GCC thinning, GCC thickness would be expected to become lower than that of the fellow eyes during follow-up. Instead, GCC thickness converged toward that of the fellow eyes, suggesting that the initially increased GCC thickness primarily reflected edema-related thickening and subsequently normalized as macular edema resolved rather than representing ongoing neuronal loss. This interpretation is further supported by the absence of a significant association between GCC thickness and final visual acuity, whereas baseline visual acuity and one-year CFT remained significant predictors of visual outcome. Taken together, our findings suggest that the longitudinal reduction in GCC thickness observed after anti-VEGF therapy predominantly reflects anatomical normalization accompanying edema resolution rather than progressive retinal neurodegeneration. Nevertheless, because diabetic retinal neurodegeneration and edema-related structural alterations may coexist in eyes with DME, longitudinal GCC changes should be interpreted cautiously, particularly when evaluating retinal neuronal damage or concomitant glaucomatous structural change. Notably, small numerical reductions in CFT, TMV, and GCC thickness were also observed in fellow eyes during the 12-month follow-up, although these changes were not statistically significant. These changes may partly reflect bilateral retinal neurodegenerative changes associated with systemic diabetes mellitus. In addition, a potential contralateral effect of intravitreally administered bevacizumab cannot be excluded. Intravitreal bevacizumab has been shown to enter the systemic circulation and reduce circulating free VEGF levels, indicating biologically relevant systemic exposure [24]. Furthermore, previous studies in patients with DME have reported reductions in macular thickness in untreated fellow eyes following unilateral intravitreal bevacizumab injections [25]. Therefore, the slight changes observed in fellow eyes in the present study may reflect the natural course of diabetes-related retinal changes, a potential systemic effect of bevacizumab, or both. However, because these changes were not statistically significant and systemic bevacizumab or VEGF levels were not measured, these possibilities remain speculative.

The relationship between inner retinal layer thickness and visual acuity in diabetic macular edema (DME) remains controversial. A previous study reported that reductions in RNFL and GCL + INL thickness in the four inner ETDRS subfields and decreases in GCL and GCL + IPL thickness in the nasal ETDRS subfields were significantly correlated with VA gain after 1 year of ranibizumab treatment [26]. In contrast, our study found no significant correlation between changes in RNFL, GCL, or IPL thickness and visual acuity improvement. This discrepancy may be partly attributable to differences in measurement methodology and analyzed retinal areas, as the previous study evaluated individual ETDRS subfields, whereas our analysis was based on average thickness within a 6 mm macular circle. Another study also demonstrated that reductions in GCL and IPL thickness within a 6 × 6 mm macular area were associated with BCVA improvement after 1 year of conbercept treatment [27]. We observed no significant correlation between either baseline or final GCC thickness and final visual acuity following 1 year of bevacizumab treatment. Differences in injection protocols may also account for these inconsistent findings, as the previous study employed three initial monthly injections followed by a pro re nata (PRN) regimen, whereas our study adopted a PRN strategy from the first injection. In our study, final visual acuity was significantly associated with baseline visual acuity and central foveal thickness at 1 year, suggesting that although visual acuity improved with the resolution of retinal edema, GCC thickness itself did not directly influence functional outcomes. From a clinical perspective, baseline visual acuity may provide useful prognostic information regarding long-term visual outcomes, while residual CFT after treatment may serve as an anatomical marker associated with final visual function. In contrast, the lack of a significant association between GCC thickness and final visual acuity suggests that GCC thinning alone may have limited value for guiding treatment decisions or predicting visual outcomes in DME. Therefore, changes in GCC thickness should be interpreted in conjunction with conventional functional and anatomical parameters, particularly visual acuity and CFT, rather than as an independent indicator of treatment response.

This study has several limitations. First, its retrospective and non-randomized design may introduce selection bias. Second, the relatively small sample size of 55 eyes warrants further research with a larger sample size to better observe changes in GCC thickness. Third, long-term studies of more than 1 year evaluating the impact of bevacizumab on GCC are needed. Fourth, we used a PRN injection regimen, so the results may differ if other regimens (e.g., T&E or fixed dosing) were applied. Fifth, all participants in this study were Korean, which may limit the generalizability of the findings to other ethnic groups. Sixth, a limitation is the use of fellow eyes as controls. Although this approach minimizes inter-individual variability, fellow eyes in patients with diabetes may not represent truly normal controls, as diabetes-related retinal neurodegeneration may occur bilaterally even in the absence of clinically apparent DME. Therefore, the differences between groups may have been underestimated. Seventh, since our study only observed changes in thickness up to the IPL, further studies exploring the impact of other retinal layers on visual acuity are warranted. Finally, standardized functional assessments such as automated perimetry were not routinely available because of the retrospective design of this study. Future prospective studies incorporating visual field testing, larger patient cohorts, and comparisons among different anti-VEGF agents may further clarify the functional significance and clinical determinants of longitudinal GCC changes in eyes with DME.

Future prospective studies with larger cohorts, longer follow-up periods, and healthy non-diabetic control groups are warranted to further distinguish edema-related changes in GCC thickness from diabetes-associated retinal neurodegeneration. Incorporating functional assessments, such as visual field testing, may help clarify the clinical significance of longitudinal GCC changes. In addition, comparisons among different anti-VEGF agents and treatment regimens, together with evaluation of bilateral retinal changes and potential contralateral effects of anti-VEGF therapy, may provide further insight into the structural and functional consequences of long-term treatment in DME.

In summary, RNFL, IPL, and GCC thicknesses were significantly greater in DME eyes at baseline compared with fellow eyes. GCC thickness significantly decreased 1 year after the first bevacizumab injection, and there was no significant difference between the DME and fellow eyes at this time. While final visual acuity was not associated with GCC thickness, baseline visual acuity and CFT at 1 year were significantly correlated with final visual acuity. These findings suggest that the observed reduction in GCC thickness after bevacizumab injection may reflect anatomical changes related to edema resolution rather than neurodegenerative processes, although further studies are needed to confirm this interpretation.

## Figures and Tables

**Table 1:** Baseline characteristics of patients with DME (n = 55).

	DME Eyes
Age (years)	53.47 ± 9.78
Male n, (%)	35 (63.6%)
DM duration (years)	11.40 ± 7.51
HbA1c (%)	8.34 ± 1.80
Lens status n, (%)	
Phakia	42 (76.4%)
Pseudophakia	13 (23.6%)
SE (diopter)	−0.60 ± 2.00
BCVA (logMAR)	0.32 ± 0.20

Values are presented as n or mean ± standard deviation. DME, diabetic macular edema; DM, diabetes mellitus; SE, spherical equivalent; BCVA, best corrected visual acuity; logMAR, logarithm of the minimal angle of resolution.

**Table 2:** Comparison of DME eyes and Non-DME fellow eyes at baseline.

	DME Eyes	Fellow Eyes	*p* Value †
Central foveal thickness, μm	440.35 ± 124.07	284.09 ± 22.62	**<0.001**
Total macular volume, mm^3^	10.89 ± 1.63	8.96 ± 0.68	**<0.001**
GCC thickness, μm	127.97 ± 19.41	109.13 ± 15.04	**0.027**
RNFL thickness, μm	49.10 ± 11.38	36.36 ± 5.48	**0.005**
GCL thickness, μm	43.10 ± 7.03	40.06 ± 6.34	0.628
IPL thickness, μm	35.77 ± 4.04	32.70 ± 4.29	**0.042**

Values are presented as mean ± standard deviation (µm or mm^3^). † Comparison between the DME and fellows eyes at initial visit. *p* value derived from the paired *t*-test. Bold font indicates statistically significant values (*p* value < 0.05). GCC, ganglion cell complex; RNFL, macular retinal nerve fiber layer; GCL, ganglion cell layer; IPL, inner plexiform layer.

**Table 3:** Comparison of DME eyes and Non-DME fellow eyes at 12 months.

	DME Eyes at Baseline	DME Eyes at 12 Months	Fellow Eyes at 12 Months	*p* Value †	*p* Value ^‡^
BCVA (logMAR)	0.32 ± 0.20	0.24 ± 0.22	0.09 ± 0.10	**0.006**	**0.073**
Central foveal thickness, μm	440.35 ± 124.07	357.51 ± 95.71	279.20 ± 24.14	**<0.001**	**<0.001**
Total macular volume, mm^3^	10.89 ± 1.63	9.79 ± 1.31	8.89 ± 0.79	**<0.001**	**0.013**
GCC thickness, μm	127.97 ± 19.41	117.09 ± 20.88	108.26 ± 17.79	**<0.001**	0.151
RNFL thickness, μm	49.10 ± 11.38	43.41 ± 10.52	36.89 ± 7.78	**<0.001**	0.102
GCL thickness, μm	43.10 ± 7.03	39.93 ± 7.34	39.05 ± 6.40	**<0.001**	0.316
IPL thickness, μm	35.77 ± 4.04	33.75 ± 5.96	32.33 ± 4.46	**<0.001**	0.153

Values are presented as mean ± standard deviation (µm or mm^3^). † Comparison between DME eyes at baseline and at the 1-year follow-up. *p* values were derived from the Wilcoxon signed-rank test. ^‡^ Comparison between the DME and fellow eyes at one year follow-up. *p* value derived from the paired *t*-test, except for IPL thickness, which was analyzed using the Wilcoxon signed-rank test because the normality assumption was not met. Baseline BCVA in fellow eyes was 0.09 ± 0.10 logMAR and remained unchanged at 12 months (*p* = 1.000). Bold font indicates statistically significant values (*p* value < 0.05). GCC, ganglion cell complex; RNFL, macular retinal nerve fiber layer; GCL, ganglion cell layer; IPL, inner plexiform layer.

**Table 4:** Final visual acuity and associated factors in DME group through multiple regression analysis.

	Final VA	
	Standardized Coefficients Beta	*p* Value
Age	0.237	0.059
Sex	0.020	0.872
Baseline VA	0.528	**<0.001**
Baseline CFT	−0.254	0.089
Baseline RNFL	0.047	0.849
Baseline GCL	0.159	0.599
Baseline IPL	−0.129	0.463
CFT at 12 months	0.326	**0.025**
RNFL at 12 months	−0.275	0.277
GCL at 12 months	−0.185	0.610
IPL at 12 months	0.148	0.564

Bold font indicates statistically significant values (*p* value < 0.05). VA, best corrected visual acuity; CFT, central foveal thickness; RNFL, macular retinal nerve fiber layer; GCL, ganglion cell layer; IPL, inner plexiform layer.

## Data Availability

The data generated and analyzed during the current study are available from the corresponding author upon reasonable request.
